# Optimization strategies for mesenchymal stem cell-based analgesia therapy: a promising therapy for pain management

**DOI:** 10.1186/s13287-024-03828-8

**Published:** 2024-07-18

**Authors:** Jing Zhang, Ping Wu, Qingping Wen

**Affiliations:** Department of Anesthesiology, The First Affiliated Hospital of Dalian Medical University, Dalian, 116000 China

**Keywords:** Mesenchymal stem cells, Pain, Analgesia therapy, Optimization strategies, Targeted delivery

## Abstract

Pain is a very common and complex medical problem that has a serious impact on individuals’ physical and mental health as well as society. Non-steroidal anti-inflammatory drugs and opioids are currently the main drugs used for pain management, but they are not effective in controlling all types of pain, and their long-term use can cause adverse effects that significantly impair patients’ quality of life. Mesenchymal stem cells (MSCs) have shown great potential in pain treatment. However, limitations such as the low proliferation rate of MSCs in vitro and low survival rate in vivo restrict their analgesic efficacy and clinical translation. In recent years, researchers have explored various innovative approaches to improve the therapeutic effectiveness of MSCs in pain treatment. This article reviews the latest research progress of MSCs in pain treatment, with a focus on methods to enhance the analgesic efficacy of MSCs, including engineering strategies to optimize the in vitro culture environment of MSCs and to improve the in vivo delivery efficiency of MSCs. We also discuss the unresolved issues to be explored in future MSCs and pain research and the challenges faced by the clinical translation of MSC therapy, aiming to promote the optimization and clinical translation of MSC-based analgesia therapy.

**Graphical Abstract:**

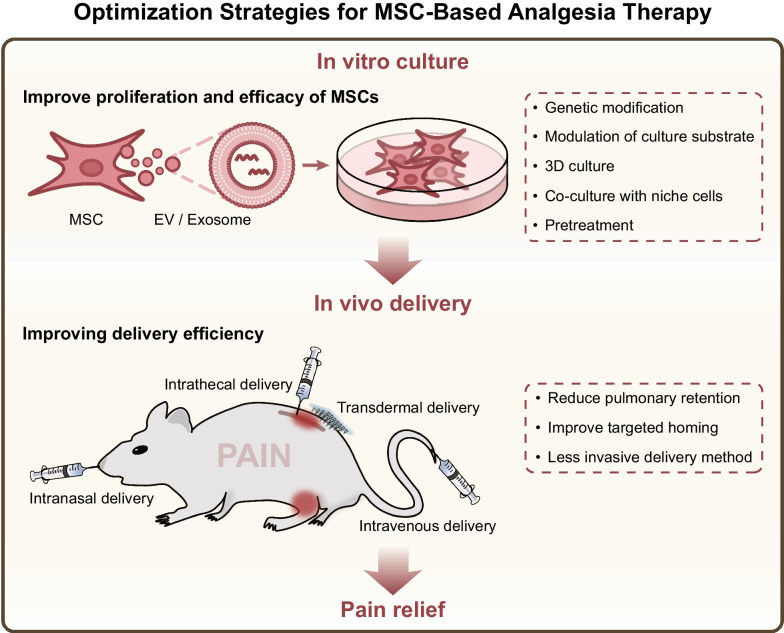

## Introduction

Pain is the most common reason for people to seek medical treatment and is the leading cause of disability worldwide. The International Association for the Study of Pain (IASP) revised the definition of pain in 2020, redefining it as an unpleasant sensory and emotional experience associated with, or resembling that associated with actual or potential tissue damage [1]. In addition to nociceptive pain caused by tissue injury and inflammation that stimulates nociceptors, and neuropathic pain caused by nerve injury, IASP introduced the term "nociplastic pain" as a third mechanistic pain descriptor and defined it as pain that arises from altered nociception despite no clear evidence of actual or threatened tissue damage causing the activation of peripheral nociceptors or evidence for disease or lesion of the somatosensory system causing the pain [1, 2]. Previous studies have shown that over 30% of people worldwide suffer from pain. Pain-induced opioid analgesic abuse, depression, and suicide seriously damaged the quality of life of patients, caused a huge personal and financial burden, and have become a major challenge to global healthcare [3].

Non-steroidal anti-inflammatory drugs (NSAIDs) and opioid analgesics are the main drugs used in clinical pain management. Adjuvant analgesics such as antidepressants and anticonvulsants are often used in combination with NSAIDs or opioids to achieve better analgesic effects. Opioids are the most effective analgesics discovered so far. The opioid-based three-step analgesic therapy remains the preferred treatment for cancer-related mixed pain [4]. However, the adverse effects of NSAIDs and opioid analgesics are the major issues limiting their clinical application and undermining their analgesic efficacy. The long-term use of NSAIDs can induce adverse reactions such as gastrointestinal ulcers, liver and kidney injury, and coagulation disorders [5]. The long-term use of opioid analgesics can cause respiratory depression, addiction, tolerance, and hyperalgesia, resulting in poor pain control and futile dose escalation [6]. Clinical data indicate that neither NSAIDs nor opioid analgesics can provide sustained and effective pain relief for patients with chronic pain, and some patients suffering from cancer-related mixed pain are not sensitive to opioid analgesics. In the face of these issues, there is currently no effective strategy to avoid the side effects of opioid analgesics, nor have any new analgesics been developed that can replace the analgesic effects of opioid analgesics. This situation is the biggest obstacle in the field of pain management, therefore, it is necessary to explore new effective analgesic strategies.

Stem cell therapy, as an emerging treatment, has shown promising efficacy in pain management. Mesenchymal stem cells (MSCs) are adult stem cells derived from the mesoderm, possessing strong proliferative and differentiation potential, as well as immunomodulatory abilities. They have a low risk of transplant rejection, which supports the use of allogeneic transplantation [7, 8]. MSCs can relieve pain through multiple mechanisms, such as alleviating neuroinflammation and inhibiting excessive activation of neurons and glial cells [9, 10]. They can also improve morphine tolerance and morphine-induced hyperalgesia [11]. MSCs have been used in the treatment of nociceptive pain, neuropathic pain, and nociplastic pain, and their effectiveness and safety have been validated in numerous preclinical studies and clinical trials [12–14]. However, unmodified MSCs have only shown moderate benefits in clinical trials. Researchers believe that this may be due to some limitations in the production, in vitro culture, and in vivo delivery of MSCs, which compromise their effectiveness [15]. Therefore, exploring strategies to enhance the analgesic efficacy of MSCs to overcome these challenges has become a focus of MSC research in recent years. With the deepening of biomaterial science research, researchers have also attempted to use biomaterials to optimize the in vitro culture environment of MSCs and promote their in vivo delivery.

Currently, there is no review comprehensively summarizing the application of MSCs in pain treatment. Therefore, this article reviews the role and advantages of MSCs in pain treatment and summarizes the recent progress in preclinical and clinical studies related to MSC-based analgesic therapies. Subsequently, we focus on the approaches and innovative methods to enhance the analgesic efficacy of MSCs, including engineering strategies to optimize the in vitro culture environment of MSCs and improve the in vivo delivery efficiency of MSCs. Finally, we discuss the unresolved questions to be explored in future MSCs and pain research, as well as the challenges of applying MSC therapy to clinical pain management, to promote the optimization and clinical translation of MSC-based analgesia therapy.

## MSC and its secretome

### MSC characteristics

MSCs are a kind of pluripotent stem cells which has powerful functions of regeneration, anti-inflammation, and immunosuppression. MSCs can be obtained from bone marrow, adipose tissue, umbilical cord, placenta, synovial fluid, synovial membrane, dental pulp, etc. So far, MSCs have shown satisfactory efficacy in preclinical animal models and clinical trials of a variety of diseases. In recent years, the role of MSCs in the treatment of pain has been gradually considered by researchers.

MSCs have the ability of self-renewal and multi-directional differentiation and can differentiate into osteoblasts, chondrocytes, adipocytes, and cardiomyocytes. Due to their strong regeneration and differentiation characteristics, MSCs are used to supplement the loss of cells to replace damaged tissue. Therefore, MSC-based cell therapy was initially widely used in the field of tissue engineering and regenerative medicine, providing an unparalleled repair strategy for tissue and organ injuries. At present, MSCs have shown effective tissue regeneration and repair effects in clinical trials of trauma, bone hypoplasia, cartilage defect, spinal cord injury, myocardial infarction, and other diseases [8]. MSCs do not express class II cell surface receptor human leukocyte antigen-DR isotype (HLA-DR) of major histocompatibility complex, so it has low immunogenicity. Preclinical studies have indicated that MSCs exhibit strong immunomodulatory effects [16–19]. MSCs can prevent anti-immune rejection in organ transplantation and significantly reduce the incidence of graft-versus-host disease (GVHD) or chronic rejection in patients undergoing hematopoietic stem cell transplantation [20]. Clinical studies have also confirmed the beneficial role of MSCs in modulating the immune system of patients undergoing liver or kidney transplantation [21, 22]. The low immunogenicity of MSCs supports their allogeneic use in clinical pain management, expanding their clinical applicability. MSCs can be administered to patients through different methods such as intravenous injection, intrathecal injection, or local delivery to the injured site. Various cytokines, chemokines, growth factors, and adhesion factors released from the injured tissue microenvironment recruit MSCs to the damaged site [23]. This process is called MSC homing and can be divided into systematic and non-systematic homing. Non-systematic homing involves directional migration of MSCs in the local or adjacent areas of the damaged tissue, while systematic homing includes multiple steps of MSCs entering the blood circulation, entering the tissue around the lesion, and finally migrating to the injured site [24]. The homing feature of MSCs enhances their effectiveness in treating various diseases. Some studies have explored strategies to enhance MSC homing to enhance the efficacy of MSCs [25].

In summary, these characteristics of MSCs determine their suitability for pain treatment. MSC-based analgesia therapy can become a promising new therapy for pain management with good clinical translational prospects.

### MSC secretome

MSC secretome includes a series of protective bioactive factors such as growth factors, cytokines, chemokines, and cell adhesion molecules, as well as lipid mediators, hormones, extracellular vesicles (EVs), and exosomes. MSC-derived EV is a lipid bilayer vesicle, which is rich in integrins, transmembrane proteins, ligands of cell surface receptors, signal transduction proteins, as well as genetic materials such as DNA, RNA, and miRNA [26]. Since the boundary of EV subclasses implied in the guidelines recommended by the International Society for EV is not clear, this review primarily focuses on well-documented microvesicles and exosomes. Microvesicles and exosomes can be distinguished by their cell origin and diameter. The diameter range of microvesicles is about 100–1000 nm, which originates from the budding of the plasma membrane. The diameter range of exosomes is about 30–200 nm, which originates from the inward budding of the late endosome membranes called multivesicular bodies (MVBs). When MVB fuses with the plasma membrane, the exosome is released into the extracellular environment and plays its biological role through a variety of mechanisms [27].

More and more studies are devoted to exploring the therapeutic role of MSC-EVs or exosomes secreted by MSCs in various diseases. The study of MSC-based “cell-free therapy” is mainly based on two considerations. On the one hand, it has been found that the therapeutic effect of MSCs is mainly mediated by MSC-EVs and a variety of cytokines secreted by MSCs [28, 29]. MSC secretome can penetrate the parenchyma of damaged tissue, rather than the MSCs themselves [30]. On the other hand, in MSC-based cell therapy, MSCs are usually cultured and expanded in vitro to obtain a sufficient number of cells. However, the long-term expansion of MSCs in vitro is accompanied by loss of cellular stemness, abnormal differentiation, and cell senescence [31]. In addition, mesenchymal stem cells transplanted into organisms are at risk of tumorigenesis and accidental differentiation. The use of MSC secretome for disease treatment is considered to be safer than the direct application of in vitro expanded MSCs. The MSC secretome lacks cellular entities that can replicate themselves, so there is no risk of tumorigenesis and accidental differentiation. Importantly, the MSC secretome does not contain the immunogenic MHC antigen found in MSCs, so the immunogenicity is low [32]. In addition, the acellular nature of the MSC secretome allows it to be aseptically filtered, thereby reducing the risk of contamination and bacterial transmission [33]. However, from the point of view of clinical transformation, the low production of EVs and exosomes has become a limiting factor for large-scale clinical applications. Therefore, future research should explore cell engineering strategies to increase the production of EVs and exosomes to realize the application of MSC secretome in clinical pain management.

## MSC-based analgesia therapy

### Advantages of MSC-based analgesia therapy

Many preclinical studies have proved that MSCs have an effective analgesic effect on nociceptive pain induced by osteoarthritis, neuropathic pain induced by nerve injury or spinal cord injury, and cancer-related mixed pain. For patients who do not respond well to traditional pain treatments, MSC therapy is an option to consider. Compared to traditional pain management methods, MSCs have unique advantages. Using MSCs alone or in combination with opioids can provide satisfactory pain relief while reducing the dosage of opioid analgesics and avoiding the risk of addiction and tolerance [11]. In contrast to neuroregulatory analgesia methods like nerve electrical stimulation, MSCs do not pose any risk of nerve injury or damage. In the treatment of osteoarthritis and discogenic pain, injecting MSCs into the articular cavity and local injured tissue can help relieve pain by repairing the damaged tissue and regulating the inflammatory microenvironment. This approach is a safe and effective alternative to surgery, as only a minimally invasive procedure is required to administer the injections. In neuropathic pain, MSCs transplanted intravenously or intrathecally homing to the site of nerve injury to facilitate nerve repair and provide analgesia. In the management of cancer-related mixed pain, intrathecal injection of MSCs is expected to replace more invasive treatments such as implantation of the intrathecal morphine pump.

### Mechanisms of MSC-based analgesia therapy

The mechanisms by which MSCs relieve pain include reducing the activation of inflammation-related pathways and the release of inflammatory factors, inhibiting the overactivation of nociceptive neurons and glial cells, and reducing hyperalgesia (Table 1). In the rat model of osteoarthritis induced by monosodium iodoacetate, intravenous and intra-articular administration of human adipose tissue-derived MSCs (hAD-MSCs) can reduce the expression of pro-inflammatory cytokines in joints by inhibiting signal transducer and activator of transcription 3 (STAT3) signaling pathway, thus improving cartilage damage and restoring osteoarthritis-related mechanical allodynia and thermal hyperalgesia [10]. In the study of neuropathic pain, it was found that early transplantation of bone marrow-derived MSCs (BM-MSCs) at day 3 after spinal cord injury (SCI) improved motor function and reduced pain hypersensitivity to mechanical and thermal stimulation. The pain analgesic effect of BM-MSCs is achieved by downregulating tumor necrosis factor alpha (TNF-α), interleukin(IL)-6 (IL-6), matrix metalloproteinase-9 (MMP-9), C–C motif chemokine ligand 2 (CCL2), CCL5, and C-X-C motif chemokine ligand 1 (CXCL1), upregulating granulocyte–macrophage colony-stimulating factor (GM-CSF), and inhibiting the expression of protein kinase C-gamma (PKC-γ) and phosphorylated cyclic adenosine monophosphate response element-binding protein (p-CREB) in neurons of the spinal cord dorsal horn. BM-MSCs can reduce the damage of the blood-spinal cord barrier (BSCB) and the recruitment of CD11b and green fluorescent protein double-positive hematogenous macrophages to the injured site. BM-MSCs significantly downregulated p-p38 mitogen-activated protein kinase (MAPK) and extracellular signal-regulated kinase (p-ERK1/2) in hematogenous macrophages and resident microglia [34]. Furthermore, intrathecal injection of BM-MSCs can relieve neuropathic pain in the mouse model of chronic constriction injury (CCI) by secreting transforming growth factor-β1 (TGF-β1), and attenuates neuropathic pain in rats with chronic compression of the dorsal root ganglion (CCD) by down-regulating purinergic receptor P2X4 (P2X4R) in spinal microglia [14, 35].

Many preclinical studies have confirmed the effectiveness of the MSC secretome in pain management. One study established a neuropathic pain model by ligating the spinal nerves of rats to explore the effect of exosomes derived from human umbilical cord MSCs (hUC-MSCs) on pain. The results showed that exosomes derived from hUC-MSCs could inhibit the activation of neurons and microglia in the spinal cord of rats, reduce the expression of inflammatory factors TNF-α and IL-1β, increase the expression of anti-inflammatory cytokines IL-10, and increase the expression of neurotrophic factors brain-derived neurotrophic factor (BDNF), glial cell line-derived neurotrophic factor (GDNF), vascular endothelial growth factor (VEGF), netrin-1 and ninjurin, which could promote nerve regeneration while anti-inflammation, thus relieving neuropathic pain in rats [36]. Another study found that exosomes from hUC-MSCs can inhibit lipopolysaccharides (LPS)-induced activation of spinal microglia and toll-like receptor 2 (TLR2)/myeloid differentiation primary response protein 88 (MyD88)/nuclear factor kappa-B (NF-κB) signal pathway by reducing the expression of radical S-adenosyl methionine domain containing 2 (Rsad2) in CCI model of chronic nerve compression injury, thus relieving neuropathic pain in rats [9]. In addition, in the mouse model of osteoarthritis-induced pain, it was found that MSC-EVs could normalize the hyperexcitability of nerve growth factor (NGF)-induced sensory neurons and significantly reduce pain-like behavior in mice [13].

Considering that miRNA carried by MSC-EVs and exosomes are important components that play a therapeutic role, some studies have explored the mechanism of the analgesic effect of miRNA secreted by MSC-EVs and exosomes. A study has found that hUC-MSCs-derived exosomes can reduce mechanical abnormal pain and thermal hyperalgesia in complete Freund’s adjuvant (CFA)-induced nociceptive pain in mice by transferring its miR-146a-5p to the dorsal horn of the spinal cord, up-regulating miR-146a-5p/tumor necrosis factor receptor-associated factor 6 (TRAF6) signaling pathway, increasing microglia autophagy and inhibiting microglial pyroptosis [37]. Another study found that MSC-EVs derived from hUC-MSCs can also transfer its miR-99b-3p to the dorsal horn of the mouse spinal cord to inhibit the activation of microglia by promoting autophagy of microglia, thereby alleviating neuropathic pain [38].

**Table 1:** Mechanisms of MSC-based analgesia therapy

MSC Type	Dose	Administration Route	Administration Time	Animals	Models	Mechanisms	Effects	References
hAD-MSCs	3 × 10^5^	Intra-articular or intravenous injection	Day 1 and 5 after osteoarthritis	Adult male Wistar rats	Osteoarthritis	Inhibited STAT3 signaling pathway, reduced expression of pro-inflammatory cytokines	Improved cartilage damage and reduced mechanical allodynia and thermal hyperalgesia	[10]
BM-MSCs	2 × 10^5^	Intraspinal injection	Day 3 post-SCI	Adult male C57BL/6N mice	SCI	Reduced the BSCB disruption and the recruitment of hematogenous macrophages, down-regulated PKC-γ, p-CREB, p-p38 MAPK, p-ERK1/2, TNF-*a*, IL-6, MMP-9, CCL2, CCL5, CXCL1 and up-regulated GM-CSF	Reduced mechanical allodynia at day 14–42 post-SCI	[34]
BM-MSCs	1 or 2.5 × 10^5^	Intrathecal injection	Day 4 or 14 post-CCI	Adult male CD1 mice	CCI	Up-regulated TGF-β1	1 or 2.5 × 10^5^ BM-MSCs injected at day 4 post-CCI reduced mechanical allodynia and thermal hyperalgesia for 5 weeks, 1 or 2.5 × 10^5^ BM-MSCs injected at day 14 post-CCI reduced mechanical allodynia and thermal hyperalgesia for 1 week or 4 weeks	[35]
BM-MSCs	1 × 10^6^	Intrathecal injection	Day 4 and 5 post-CCD	Adult male Wistar rats	CCD	Down-regulated P2X4R	Reduced mechanical allodynia transiently at day 6 post-CCD	[14]
hUC-MSCs-derived exosomes	5 μg	Intrathecal injection	Day 1–7 after surgery, once a day	Adult male Sprague–Dawley rats	Spinal nerve ligation	Down-regulated c-Fos, GFAP, Iba-1, TNF-α, and IL-1β, up-regulated IL-10, BDNF, and GDNF	Reduced mechanical allodynia and thermal hyperalgesia for 7 days after exosomes injection	[36]
hUC-MSCs-derived exosomes	5 μg	Intrathecal injection	Day 2, 4 and 6 post-CCI	Adult male Sprague–Dawley rats	CCI	Down-regulated Rsad2, inhibited activation of the TLR2/MyD88/NF-κB signaling pathway and microglia	Reduced mechanical allodynia for 7 days after exosomes injection	[9]
MSCs or MSC‐EVs	2 × 10^4^ MSCs or MSC‐EVs derived from 2 × 10^4^ MSCs	Intra-articular injection	14 weeks after osteoarthritis	Adult male C57BL/6 J mice	Osteoarthritis	Inhibited NGF-induced sensory neuron hyperexcitability	Reduced pain at 14–16 weeks after osteoarthritis	[13]
hUC-MSCs-derived exosomes	5 µg	Intrathecal injection	Day 0–4 after CFA injection, once a day	Adult male C57BL/6 J mice	CFA-induced nociceptive pain	Transferred miR-146a-5p to spinal cord, activated miR-146a-5p/TRAF6 signaling pathway, promoted microglia autophagy and inhibited microglial pyroptosis	Reduced mechanical allodynia and thermal hyperalgesia at day 1–7 after CFA injection	[37]
miR-9-5p modified BM-MSCs	6 × 10^6^	Intrathecal injection	From 2 days before BCP to day 21 post-BCP, once a day	Adult male C3H/HeN mice	BCP (NCTC murine sarcoma cells)	Down-regulated REST, TNF-α, IL-6, IL-1β and up-regulated MOR	Reduced mechanical allodynia at day 7–21 after BCP operation	[12]
hPPE modified hBM-MSCs	6 × 10^6^	Intrathecal injection	Day 11 post-BCP	Adult female Sprague–Dawley rats	BCP (Walker 256 mammary gland carcinoma cells)	Down-regulated IL-1β and IL-6	Reduced mechanical allodynia at day 12–21 after BCP operation	[39]

To sum up, preclinical studies have shown that MSC-EVs and exosomes can effectively contribute to pain treatment. Further preclinical studies will continue exploring the potential molecular mechanisms of MSCs in providing analgesic effects. This research will provide theoretical support and guidance for clinical applications of MSCs.

### Clinical trials of MSC-based analgesia therapy

According to the records of ClinicalTrials.gov, as of April 14, 2024, we summarized 28 interventional clinical trials of MSC-based analgesia therapy with pain as the outcome measure (Table 2). In these clinical trials, discogenic pain and joint-related pain accounted for the majority, while there were 2 trials of pancreatitis pain, and one each for refractory migraine and trigeminal neuralgia. It can be seen that currently, clinical trials on MSC-based pain therapies are mainly focused on neuropathic pain and nociceptive pain. There are currently no clinical trials on using MSCs for the treatment of cancer-related mixed pain, mainly due to concerns about the safety of MSCs in cancer patients. Many preclinical studies on MSC therapy for cancer-related mixed pain have inconsistent conclusions regarding whether MSCs have potential pro-cancer effects. The discrepancies could be attributed to the differences in animal models, cancer cell lines, MSC doses, and delivery routes used in the experiments. Therefore, preclinical experiments in the future need a more comprehensive, systematic, and long-term assessment of the safety of using MSCs to treat cancer-related mixed pain. More importantly, when conducting clinical trials for cancer-related mixed pain in the future, the lowest effective dose of MSCs should be used, and the delivery route should be selected reasonably, with long-term follow-up of the participants.

**Table 2:** Clinical trials of MSC-based analgesia therapy

Trial ID	Status	Conditions	Interventions	Enrollment	Phases	Start Date	Locations	Study Results
NCT01860417	Completed	Discogenic low back pain	Intradiscal injection of hBM-MSCs	25	Phase 1|Phase 2	01/04/2013	Hospital Clinico Universitario, Spain	No results posted
NCT02097862	Completed	Discogenic low back pain	Intradiscal injection of hAD-MSCs	15	NA	01/03/2014	US Stem Cell Clinic, United States	No results posted
NCT01739504	Terminated	Osteoarthritis Pain	Intra-articular injection of hAD-MSCs	10	NA	01/03/2014	Ageless Institute LLC, United States	No results posted
NCT02338271	Unknown status	Discogenic low back pain	Intradiscal injection of hUC-MSCs	10	Phase 1	01/01/2015	CHA University, Korea	No results posted
NCT02958267	Completed	Knee Osteoarthritis Pain	Intra-articular injection of hBM-MSCs	32	Phase 2	01/12/2016	McConnell Spine, Sport, and Joint Physicians, United States	Has Results
NCT04499105	Unknown status	Discogenic low back pain	Intradiscal injection of hUC-MSCs	10	Phase 2	24/07/2017	Cipto Mangunkusumo Hospital, Indonesia	No results posted
NCT03337243	Completed	Knee Osteoarthritis Pain	Intra-articular injection of hUC-MSCs	60	NA	09/11/2017	Scott Medical Health Center, United States	No results posted
NCT04064879	Suspended	Refractory Migraine	Intravenous, intra-articular, and soft tissue injection of hAD-MSCs	10	NA	16/08/2018	Neurological Associates of West Los Angeles, United States	No results posted
NCT03477942	Recruiting	Knee Osteoarthritis Pain	Intra-articular injection of hBM-MSCs	16	Phase 1	01/10/2018	University Hospital Cleveland Medical Center, United States	No results posted
NCT03608579	Recruiting	Hip Osteoarthritis Pain	Intra-articular injection of hAD-MSCs	24	Phase 1	05/11/2018	Mayo Clinic in Rochester, United States	No results posted
NCT04104412	Recruiting	Discogenic low back pain	Intradiscal injection of hUC-MSCs	242	Phase 1	15/08/2019	Xuanwu Hospital Capital Medical University, China	No results posted
NCT04410731	Active, not recruiting	Pain of lumbar facet arthropathy	Intra-articular injection of hBM-MSCs	10	Phase 1	28/04/2020	Mayo Clinic in Florida, United States	No results posted
NCT05018637	Unknown status	Pain of vertebral compression fracture	Intramedullary injection of hUC-MSCs in Combination subcutaneous injection of teriparatide	30	Phase 2	01/09/2020	CHA University, Korea	No results posted
NCT04530071	Completed	Discogenic low back pain	Intradiscal injection of hUC-MSCs	36	Phase 1|Phase 2	21/09/2020	CHA Bundand Medical Center, Korea	No results posted
NCT04759105	Active, not recruiting	Discogenic low back pain	Intradiscal injection of hBM-MSCs	48	Phase 2	17/11/2020	Fondazione IRCCS Ca’ Granda, Italy	No results posted
NCT05066334	Unknown status	Discogenic low back pain	Intradiscal injection of hBM-MSCs	52	Phase 2	22/03/2021	Campus Bio-Medico University of Rome, Italy	No results posted
NCT05011474	Unknown status	Discogenic low back pain	Intradiscal injection of Matrillin-3 pretreated hAD-MSCs	4	Phase 1|Phase 2	23/04/2021	CHA University, Korea	No results posted
NCT05305833	Unknown status	Pain of temporomandibular joint disorders	Intra-articular injection of hUC-MSCs	20	Phase 1|Phase 2	01/10/2021	Erciyes University, Turkey	No results posted
NCT03390920	Not yet recruiting	Pain of all types of musculoskeletal conditions	Injection of hUC-MSCs	200	NA	01/01/2022	Advanced Stem Cell Institute, United States	No results posted
NCT05261360	Recruiting	Pain of degenerative meniscal injury	Intra-articular injection of human synovial fluid-derived MSCs (hSF-MSCs)	30	Phase 2	01/03/2022	Eskisehir Osmangazi University, Turkey	No results posted
NCT05288725	Not yet recruiting	Knee Osteoarthritis Pain	Intra-articular injection of hBM-MSCs	120	Phase 1|Phase 2	01/08/2022	Bluetail Medical Group, United States	No results posted
NCT05152368	Recruiting	Trigeminal neuralgia	Intravenous injection of hUC-MSCs	20	Phase 1	09/09/2022	Medical Surgical Associates Center, Antigua and Barbuda	No results posted
NCT05783154	Recruiting	Knee Osteoarthritis Pain	Intra-articular injection of hAD-MSCs	84	Phase 2|Phase 3	16/09/2022	Bangabandhu Sheikh Mujib Medical University, Bangladesh	No results posted
NCT05815771	Not yet recruiting	Pain of temporomandibular joint disorders	Intra-articular injection of hBM-MSCs	24	NA	01/07/2023	Cairo university, Egypt	No results posted
NCT06001853	Recruiting	Pain of lumbar facet arthropathy	Intra-articular injection of hBM-MSCs	40	Phase 2	03/11/2023	Mayo Clinic in Florida, United States	No results posted
NCT05925036	Recruiting	Pain of chronic pancreatitis	Intravenous injection of hBM-MSCs	40	Phase 1	01/01/2024	Ralph H. Johnson VA Medical Center, United States	No results posted
NCT06205342	Not yet recruiting	Pain of chronic pancreatitis	Intravenous injection of hBM-MSCs	48	Early Phase 1	01/05/2024	Medical University of South Carolina, United States	No results posted
NCT04735185	Suspended	Discogenic low back pain	Intradiscal injection of hBM-MSCs	106	NA	30/09/2025	Johns Hopkins Hospital, United States	No results posted

## Optimization strategies for MSC-based analgesia therapy

### *Strategies for improving the *in vitro* proliferation and efficacy of MSCs*

**Fig. 1:**
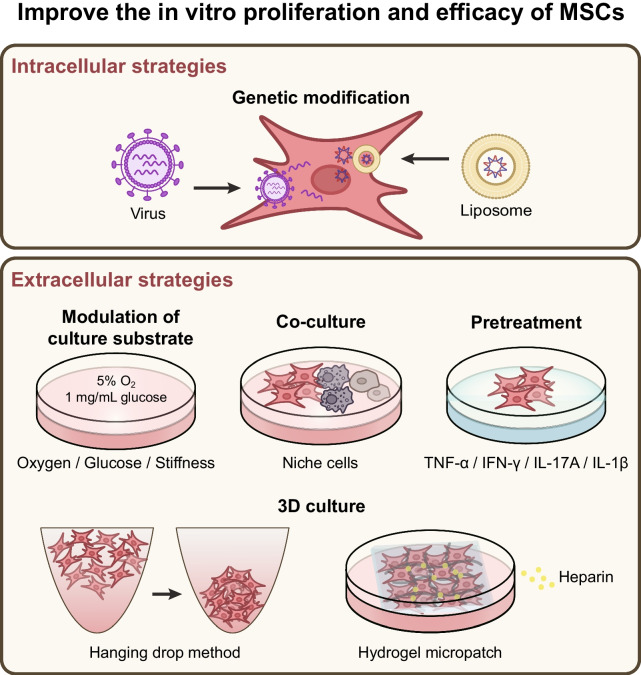
Strategies for improving the in vitro proliferation and efficacy of MSCs

MSCs have been used to treat a wide variety of diseases. However, the quantity of MSCs that can be extracted from tissues is often limited and insufficient for disease treatment purposes. Therefore, it is necessary to culture and expand the extracted MSCs in vitro in order to obtain a sufficient number of cells for subsequent applications. The term “stem cell niche” refers to the specific environment in which stem cells exist. This environment consists of MSCs, non-stem cells in the surrounding area, extracellular matrix (ECM), cell adhesion molecules, soluble factors, vascular networks, and nerve fibers. In vivo, these components work together in a complex and dynamic manner to regulate the activity of MSCs and determine their fate [40]. The stemness properties, surface markers, transcriptional spectra, and differentiation characteristics of MSCs can change during in vitro culture due to the disparity between the in vitro culture environment and the in vivo microenvironment. These changes ultimately reduce the proliferation rate of MSCs in vitro and impact their survival rate and therapeutic effectiveness after in vivo transplantation [41, 42]. Therefore, various strategies, including transforming MSCs and optimizing their culture environments, have been developed to maintain the original characteristics and functions of MSCs while promoting their proliferation in vitro (Fig. 1).

#### Intracellular engineering strategies

Intracellular engineering strategies primarily involve genetic modification and epigenetic modification. Gene modification is a genetic engineering technique that modifies DNA sequence by biochemical methods. The gene modification system can change the genome and gene expression of target cells by inserting, deleting, or modifying genes, to regulate the functional characteristics of cells. Currently, gene modification methods include viral vector-based gene modification systems consisting of lentiviruses, retroviruses, adenoviruses, and adeno-associated viruses, and non-viral gene modification systems such as DNA, RNA, protein, and protein-RNA complexes [43]. Epigenetic regulation refers to heritable changes in gene function through modifications to the chromatin state, without changing the DNA sequence of the gene. The regulatory mechanisms of epigenetics include DNA modification, histone modification, and regulation of non-coding RNA [44]. The strategy of gene modification or epigenetic modification of MSCs has been widely used in preclinical research. By modulating the expression of specific genes in MSCs, it is possible to increase the proliferation ability of MSCs, control their differentiation tendency, and improve their therapeutic efficacy in various diseases [45–49]. In addition, as a direct gene editing technology, CRISPR/Cas9 is a new genetic modification tool with simple operation and high efficiency in gene editing [50]. The clustered regularly interspaced short palindromic repeats (CRISPR)-associated protein 9 (Cas9) specifically cleaves the target DNA sequence to produce DNA double-strand breaks. Subsequently, the intracellular DNA repair system repairs the double-strand breaks and changes the DNA sequence during the repair process [51, 52]. Many studies have utilized CRISPR/Cas9 to modulate the gene expression of MSCs with the aim of augmenting the therapeutic efficacy of MSCs in conditions such as diabetes, osteoarthritis, and other diseases [53–55].

The intracellular engineering strategies of MSCs have been used for pain management. The μ-opioid receptor (MOR) plays a crucial role in the signaling pathway of pain, and its expression can be inhibited by repressor element-1 silencing transcription factor (REST) [56–58]. It is reported that miR-9-5p secreted by BM-MSCs can regulate the expression of REST and MOR. Therefore, a study upregulated miR-9-5p in BM-MSCs using a lentiviral vector to investigate the effect of miR-9-5p modified BM-MSCs on bone cancer pain. The results showed that intrathecal injection of miR-9-5p modified BM-MSCs could reduce the expression of REST and increase the expression of MOR, inhibit the release of inflammatory factors TNF-α, IL-6, and IL-1β in the spinal cord dorsal horn of mice, and relieve bone cancer pain [12]. In addition, in another study, the researchers modified hBM-MSCs with the human proenkephalin (hPPE) gene, which has an analgesic effect [59]. This study found that intrathecal injection of hPPE-modified hBM-MSCs could effectively relieve bone cancer pain in rats by inhibiting the expression of pro-inflammatory cytokines, including IL-1β and IL-6 [39]. Furthermore, GDNF, IL-10, and TGF-β are the analgesic factors in the secretome of MSCs. Increasing the expression of these analgesic proteins in MSCs by viral transduction can enhance the analgesic efficacy of MSCs in pain treatment [60]. Therefore, these studies demonstrate the potential role of genetic engineering in pain management.

However, it is worth noting that while genetic modification and epigenetic modification have been widely used in preclinical research for many diseases, they have some limitations. Due to the lack of specificity, these modification strategies may accidentally change the DNA sequence of non-target regions, resulting in gene mutations or cytotoxicity [61]. Hence, the clinical applications of genetic modification and epigenetic modification are limited by public concerns regarding ethical and safety issues.

#### Extracellular engineering strategies

The extracellular engineering strategies aim to modify the in vitro culture environment to mimic the in vivo niche of MSCs, or to pretreat MSCs to enhance its functional properties. These methods are considered safer and more feasible than genetic modification. In recent years, new strategies have emerged, including the regulation of oxygen and glucose in the culture environment, the construction of the three-dimensional (3D) culture environment, the optimization of substrate stiffness, the co-culture of MSCs and other niche cells, and the pretreatment of MSCs.

##### Modulation of oxygen and glucose in the culture environment

MSCs reside in microenvironments in vivo with low oxygen concentrations ranging from 1 to 15%, depending on their tissue sources. The physiological oxygen concentration in vivo is significantly lower than the commonly used 21% oxygen concentration in the laboratory culture of MSCs under normoxic conditions [62]. Research has shown that high oxygen concentration in the culture environment can cause DNA damage and accelerate senescence in MSCs by generating more reactive oxygen species (ROS) [63]. Therefore, it is recommended to apply hypoxia to the culture environment of MSCs to regulate their proliferation and metabolism in vitro. Hypoxia, as a physiological stimulus, can activate intracellular signaling pathways and promote cell adaptation [64]. A recent study demonstrated that exposure of MSCs to moderate hypoxia with a 5% oxygen concentration resulted in increased proliferation rate, shorter doubling time, and metabolic hyperactivity, which depended on oxidative phosphorylation and glycolysis. In contrast, when MSCs were exposed to severe hypoxia with a 1% oxygen concentration, their proliferation was stagnant, and they exhibited low metabolism dependent on anaerobic glycolysis [65]. These findings suggest that moderate hypoxia with approximately 5% oxygen concentration is an ideal condition for culturing MSCs in vitro.

The growth and differentiation of MSCs are significantly influenced by the glucose levels in their surrounding environment. Hormones such as insulin and glucagon play a vital role in regulating blood glucose levels in healthy individuals, maintaining it at around 1 mg/mL. When MSCs are exposed to a high glucose medium of 4.5 mg/mL, it results in significant inhibition of proliferation and cell senescence [66]. Additionally, a high glucose medium promotes osteogenic differentiation but weakens the chondrogenic capacity of MSCs [67]. Thus, when culturing MSCs in vitro, it is crucial to maintain a glucose level similar to that of their natural niche.

##### Construction of the 3D culture environment

The in vivo niche of MSCs is a microenvironment with a 3D structure, which provides adhesion sites and physical support for MSCs so that MSCs can interact with each other. In addition to providing a suitable dimension for MSCs, ECM in the niche is rich in collagen, fibronectin, laminin, and other matrix proteins. The chemical signals transmitted by these proteins regulate the activity and function of MSCs [68, 69]. In the two-dimensional (2D) plane culture environment in vitro, due to the lack of the 3D environment and matrix proteins provided by ECM, MSCs lose their signal connection with other cells and proteins. This is also one of the main reasons for the low survival rate and proliferation rate of MSCs in vitro. At present, researchers have designed many strategies to simulate the 3D culture environment of MSCs in vivo. These strategies include extracting natural ECM from vivo, forming spheres of MSCs by hanging drop culture plates and low adhesion plane technology, and using biomaterials such as hydrogel to construct 3D scaffolds [70–72]. The natural ECM extracted from the body tissue contains a complete ECM protein, which better simulates the ECM environment in vivo [73]. The strategy of MSCs spheroid culture and using biomaterials to construct the 3D culture environment requires the addition of ECM matrix proteins, and it is worth noting that complete ECM matrix proteins should be added instead of a single matrix protein [72, 74].

The 3D culture environment affects the morphology, proliferation, and differentiation of MSCs. Compared to MSCs cultured in a 2D environment, 3D-cultured MSCs show a spindle-shaped or spherical shape, a higher proliferation rate, and a stronger ability for multi-lineage differentiation [75]. In various inflammatory diseases, 3D-cultured MSCs showed stronger anti-inflammatory ability and better therapeutic efficacy than 2D-cultured MSCs [76, 77]. The 3D-culture strategy of MSCs has also shown excellent effects in pain management. Some studies have found that in the neuropathic pain model of sciatic nerve ligation, the in vivo survival rate of tonsil-derived MSC (T-MSC) spheroids cultured by the hanging drop method is higher than that of monolayer 2D T-MSCs. T-MSC spheroids can effectively relieve the pain of mice by significantly inhibiting the expression of inflammatory factors TNF-α and interferon-γ (IFN-γ) and the infiltration of macrophages into the injured tissue [78]. In addition, in another study of neuropathic pain induced by sciatic nerve injury, researchers created a heparin-based hydrogel micropatches loading with hAD-MSCs. The heparin provides adhesion sites for hAD-MSCs, while the hydrogel provides physical support. The heparin-based hydrogel micropatches can promote the proliferation and maintain the stemness of hAD-MSCs in vitro. Furthermore, they can improve the survival rate and retention rate of hAD-MSCs in injured nerve tissue, and enhance the therapeutic effect of hAD-MSCs in nerve repair and pain relief [79].

##### Optimization of culture substrate stiffness

The characteristics and behavior of MSCs are regulated by the physical properties of the in vivo niche. For MSCs cultured in vitro, the stiffness of the culture substrate is an important physical characteristic to control the fate of MSCs [80]. A rigid matrix can improve the proliferation ability and promote osteogenic differentiation of MSC, while a soft matrix is beneficial to adipogenic differentiation [81]. MSCs cultured in a soft matrix have a stronger immunomodulatory effect and can significantly reduce macrophage-mediated inflammatory response in vivo. The immunomodulatory effect induced by soft matrix is mainly related to the promotion of TNF-α-stimulated gene 6 (TSG-6) expression in MSCs [82]. Therefore, adjusting the stiffness of the culture substrate is helpful to enhance the anti-inflammatory effect of MSCs in vivo. This strategy is expected to be used in the culture of MSCs in vitro and the MSC-based therapies for pain.

##### Co-culture of MSCs and other niche cells

The niche of MSCs is a complex environment. In addition to MSCs, there are many different types of non-stem cells, including macrophages, hematopoietic stem cells, endothelial cells, osteoblasts, adipocytes, and nerve cells. The close interaction between MSCs and these cells affects the proliferation and differentiation of MSCs. In the niche of MSCs, MSCs interact with macrophages through intercellular contact, secretion of soluble factors, and organelle transfer. Macrophages can regulate the proliferation and differentiation of MSCs. M2 macrophages promote the proliferation of MSCs, while M1 macrophages induce MSC apoptosis [83]. Macrophages in various polarized states, including M1 and M2, can inhibit the adipogenic differentiation of MSCs, while M1 macrophages promote the osteogenic differentiation of MSCs [84, 85]. In addition, it has been found that MSC-macrophage crosstalk plays an important role in maintaining the homeostasis of the inflammatory microenvironment. The intercellular contact between MSCs and M1 macrophages enhanced the inhibitory regulation of MSCs on T cells and promoted the transformation of macrophages to anti-inflammatory phenotype [86]. IL-8, CCL2, and CCL5 secreted by macrophages can induce high expression of TSG-6, CCL5, and CXCL10 in MSCs, and increase its anti-inflammatory and migration ability in inflammation and tissue injury response in vivo [86, 87]. Therefore, the co-culture of macrophages and other cells in MSCs in vitro can enhance the proliferation and anti-inflammatory function of MSCs. Given that neuroinflammation is an important participant in the mechanism of pain, MSCs co-cultured with macrophages in vitro may be more effective in inhibiting inflammatory response and relieving pain.

##### Pretreatment of MSCs

Neuroinflammation is a crucial component of the central mechanism of various types of pain. Neurons and glial cells activated in the spinal cord and brain secrete large amounts of inflammatory factors, creating an inflammatory microenvironment [88]. The inhibition of neuroinflammation by MSCs is a key mechanism through which they exert analgesic effects. This neuroinflammatory environment can affect the in vivo survival and immune regulatory abilities of MSCs. The immune regulatory ability of MSCs is not constitutive but induced by inflammatory cytokines. Only when MSCs are exposed to sufficiently high levels of pro-inflammatory cytokines can they exert immunosuppressive effects. The activation level of MSCs may vary depending on the level and type of inflammation in the resident tissue [89]. TNF-α and IFN-γ are key inflammatory factors released by immune cells during inflammation. In in vitro cell experiments, TNF-α or IFN-γ are often used to simulate the occurrence and development of the inflammatory environment in the body. Studies have found that pretreatment of MSCs with TNF-α and IFN-γ can enhance their immune regulatory abilities [90]. TNF-α or IFN-γ pretreated MSCs exhibit significantly enhanced regulatory abilities in T helper 1 cell (Th1)/Th2 responses and macrophage M1/M2 polarization in inflammatory environments [91, 92]. Furthermore, MSCs pretreated with IL-17A and IL-1β have also been found to have this enhancing effect [93, 94]. These pretreatment strategies aim to enhance the anti-inflammatory and immune regulatory capabilities of MSCs in inflammatory environments, as well as bolster their resilience against the impacts of such environments. Therefore, many current strategies aim to specifically pretreat MSCs with the consideration of the host environment in which they are transplanted, to maximize their efficacy.

In summary, the survival, proliferation, and functional properties of MSCs will be influenced by genetic modification, pretreatment, and in vitro culture conditions. Efforts should be made to consider the mechanisms and characteristics of pain as much as possible and to modify MSCs and their cultural environment in a targeted manner. This will help stimulate the optimal analgesic efficacy of MSCs.

### *Strategies for improving the *in vivo* delivery efficiency of MSCs*

After achieving high-level expansion of MSCs in vitro through various cell engineering strategies, the next step is to select a suitable method of administration to deliver MSCs to the target site in the body. The occurrence of pain is based on the activation status of various cells in the nervous system and changes in signaling networks. Drugs and cells used for pain management must target the tissues and structures involved in pain signal transmission pathways, such as the dorsal root ganglia, spinal cord, and brain [36, 95]. In current preclinical studies, MSCs are primarily used for pain management in experimental animals through intravenous injection, intrathecal injection, and intranasal administration. These methods of MSC administration deliver MSCs to the dorsal root ganglia, spinal cord, and brain through various delivery mechanisms, effectively alleviating pain in different pain models such as neuropathic pain, nociceptive pain, and nociplastic pain in experimental animals. MSCs administered through intravenous injection rely on their homing characteristics to migrate to the specific spinal cord segment where pain occurs [24]. In contrast, MSCs administered through intrathecal and intranasal injection can directly contact the spinal cord by utilizing cerebrospinal fluid circulation and substance transport within neurons. This mechanism allows for more direct and potent analgesic effects [35, 96].

**Fig. 2:**
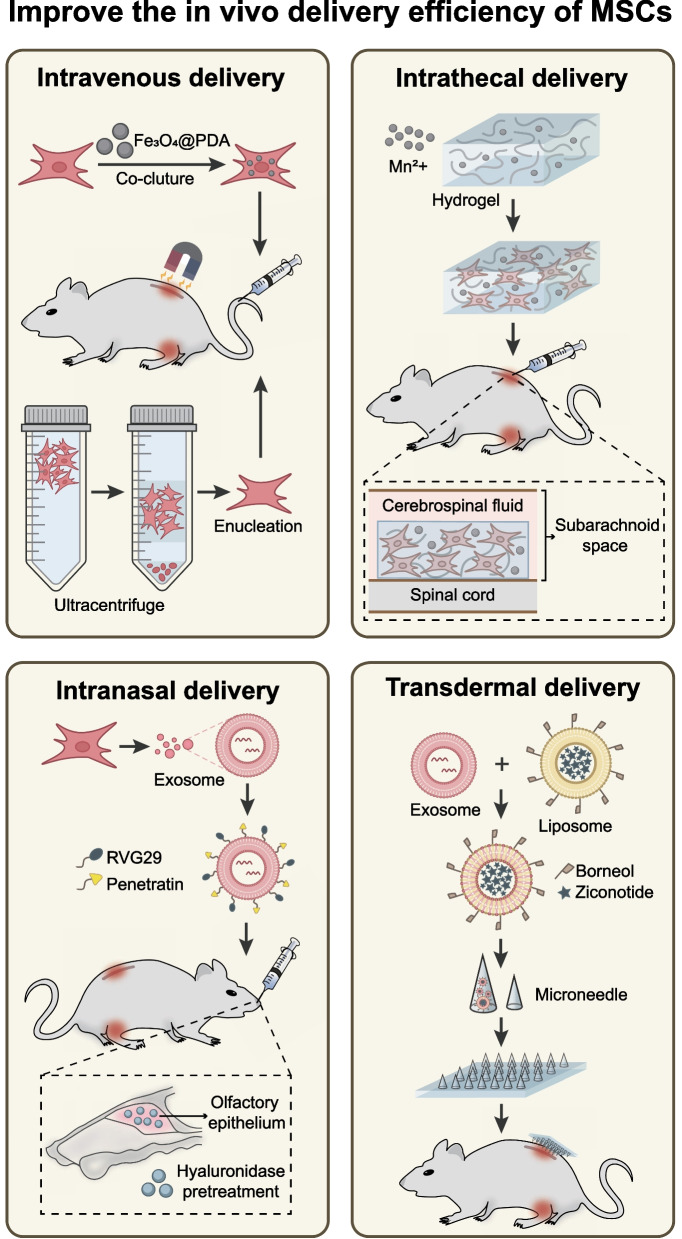
Strategies for improving the in vivo delivery efficiency of MSCs

The efficacy of MSCs in preclinical pain research provides ample evidence and theoretical support for their clinical translation. It should be noted that in clinical practice, the method of administering MSCs to patients needs to consider potential complications and safety risks. These three methods of MSC delivery, namely intravenous injection, intrathecal injection, and intranasal administration, each have their own advantages and disadvantages for patients, as well as different safety risks. The optimal method of intrathecal injection for analgesia in experimental animals may result in various complications in clinical patients, such as spinal cord injury and infection. Intranasal administration and intravenous injection, as minimally invasive drug delivery methods, have drawbacks such as low homing efficiency and poor targeting. In recent years, many studies have utilized biomaterials and cell engineering techniques to optimize the in vivo delivery of MSCs for enhancing the clinical translation of MSC-based analgesia therapy. These strategies aim to address various drawbacks of different MSC delivery methods and enhance targeted delivery efficiency and analgesic effects of MSCs (Fig. 2).

#### Intravenous delivery

Intravenous injection is the most commonly used method of drug administration in clinical practice. Intravenous injection is widely accepted by patients because of its advantages, including minimal trauma, ease of implementation, and the absence of need for specific tools and equipment. It is also suitable for long-term, continuous, or multiple drug infusions. MSCs have the ability to migrate preferentially to the injured site, a characteristic known as homing. Ischemic, hypoxic, or injured tissues express a variety of signaling molecules, such as chemokines and adhesion molecules, which attract MSCs to migrate toward blood vessels and colonize the target tissue through vascular endothelial cells [24]. Due to this characteristic, MSCs have played a significant role in the treatment of many diseases. It is worth noting that many preclinical studies have found that the homing rate of MSCs is low when they are injected intravenously to treat pain in experimental animals. MSCs injected intravenously into the body must first pass through the pulmonary circulation [97]. In some studies, an in vivo imaging technique was used to monitor the fate of MSCs injected into the tail vein of mice. Most of the MSCs labeled with fluorescence were found to be trapped in the lungs of mice. Over time, some of the MSCs stranded in the lungs will detach and migrate to the damaged tissue [98]. However, this process prolongs the time needed to achieve the desired therapeutic effect and reduces its effectiveness. The diameter of the smallest capillary in the human lung is 6–9 μm, allowing only substances smaller than 5 μm to pass through smoothly. In contrast, the diameter of MSCs ranges from 15 to 25 μm. Therefore, this obstacle also exists when intravenous MSCs are used in clinical treatment. The diameters of extracellular vesicles and exosomes released by MSCs are 0.1–1 μm and 0.05–0.15 μm, respectively [99]. They can pass through the pulmonary capillary intima smoothly, making them a popular alternative to MSCs in the treatment of various diseases. In addition, the blood–brain barrier is a crucial factor that prevents many drugs for the nervous system from being administered intravenously. Intravenous MSCs are unable to pass through the blood–brain barrier. Many preclinical studies have observed the regulatory effect of MSCs on spinal cord neurons and glial cells in pain. This effect may be achieved through the secretion of cytokines, as well as the release of extracellular vesicles and exosomes [100]. Extracellular vesicles and exosomes have the ability to traverse the blood–brain barrier, making them valuable tools for delivering drugs to the brain. Therefore, the primary obstacle to the application of MSCs for pain treatment is the limited effectiveness of MSCs homing to the spinal cord. The primary challenge that requires urgent attention is how to overcome the dual limitations of pulmonary capillaries and the blood–brain barrier to improve the effective homing of MSCs or their extracellular vesicles and exosomes for pain management.

Enhancing the targeting of MSCs to injured tissues can improve efficacy and reduce the side effects associated with the retention in other tissues and organs. To improve the targeting capability of MSCs in the treatment of neuropathic pain, a study developed composite superparticles Fe_3_O_4_@polydopamine (PDA) by using iron oxide nanoparticles (NPs) in the form of Fe_3_O_4_ as the core and PDA as the shell. The superparticles were then combined with MSCs to create Fe_3_O_4_@PDA-labeled MSCs. Superparamagnetic Fe_3_O_4_ nanoparticles are biocompatible and commonly used as contrast agents for magnetic resonance imaging in clinical settings to diagnose cancer [101]. The PDA coating is formed through the spontaneous polymerization of dopamine (DA). This coating demonstrates excellent biocompatibility and biodegradability [102]. After injecting Fe_3_O_4_@PDA-labeled MSCs into rats with chronic compression sciatic nerve injury (CCI) via the caudal vein, a magnetic field was used to guide the MSCs to the injured segment of the spinal cord. This process promotes the homing and aggregation, ultimately enhancing spinal cord repair and alleviating pain. It has been found that Fe_3_O_4_@PDA does not affect the characteristics or viability of MSCs. Fe_3_O_4_@PDA-labeled MSCs exhibited increased migration to the spinal cord with minimal retention in the lungs 24 h after intravenous injection, while the majority of unlabeled MSCs remained in the lungs. In addition, Fe_3_O_4_@PDA-labeled MSCs reduced spinal nerve demyelination and the expression of the pain-related factor c-Fos. They also inhibited the activation of microglia and astrocytes, and generated an immediate and long-lasting analgesic effect in neuropathic pain [103]. These results demonstrate the safety and feasibility of this MSC labeling strategy in enhancing the targeting effect of MSCs in vivo. Furthermore, it serves as a reference for the development of MSC engineering strategies and their application in clinical neuropathic pain patients.

In another study, to overcome the barrier of pulmonary capillaries and deliver MSCs, the nucleus of MSCs was removed using density gradient centrifugation. Enucleated MSCs exhibited a smaller cell volume and improved deformability, allowing them to pass through 5 μm pores in vitro. In the in vivo study, enucleated MSCs injected into mice with acute ear inflammation and acute pancreatitis through the tail vein showed a lower pulmonary retention rate and a higher homing rate to the injured tissue compared to untreated MSCs in vivo imaging systems. The enucleated MSCs effectively reduced inflammation and tissue injury. Given that a variety of chemokines and integrins are expressed at the injured site, enhancing the expression of chemokine receptors and integrin receptors in MSCs through genetic modification can increase their ability to migrate to the injured tissue [104]. These findings suggest that nuclear removal can be an effective strategy for improving the homing rate of MSCs. More importantly, enucleated MSCs lose the ability to proliferate, which prevents the risk of abnormal differentiation and tumor formation of MSCs in vivo, ensuring the safety of their application in clinical pain management. In addition, enucleated MSCs have acquired the ability to carry drugs, making them suitable as cellular carriers for delivering analgesic drugs.

#### Intrathecal delivery

The intrathecal injection can deliver MSCs directly to the central nervous system without being hindered by the blood–brain barrier. MSCs injected into the subarachnoid space were intended to migrate to the injured site of the central nervous system via the cerebrospinal fluid. Hence, the intrathecal injection of MSCs can produce a faster and more potent analgesic effect compared to intravenous injection. However, as an invasive method of drug administration, intrathecal injection and intrathecal catheter implantation not only have many clinical contraindications but also carry the risk of complications such as intraspinal hemorrhage, hematoma, infection, nerve injury, and catheter blockage. As a result, the acceptance of intrathecal injection is much lower than that of intravenous injection. In addition to the well-known clinical risks of intrathecal injection, researchers have gradually noticed some limitations of using MSCs to treat pain through intrathecal injection. Because the MSCs injected into the sheath can move freely in the cerebrospinal fluid, the postural changes accompanied by spontaneous movement of experimental animals or patients will result in continuous repositioning of the injected MSCs within the sheath due to the influence of gravity. For example, when humans stand or sit, the cells often settle around the cauda equina below the spinal cord [105]. Therefore, the retention rate of MSCs in the targeted spinal cord segment is very low. Furthermore, research has shown that intrathecal injection of MSCs leads to the spontaneous formation of aggregates in cerebrospinal fluid and results in ventricular dilatation in rats. The high expression of Integrin α4 and vascular cell adhesion molecule-1 (VCAM-1), along with their interaction, may be the primary factors promoting MSC aggregation [106]. Given the tendency of mesenchymal stem cells (MSCs) to aggregate spontaneously, it is crucial to fully consider the risk of obstructing the flow of cerebrospinal fluid when administering a high dose of MSCs into the sheath of patients experiencing clinical pain.

Due to these limitations, many studies have focused on developing engineering techniques to incorporate MSCs into biomaterials. This aims to tackle the challenges of inadequate cell deposition and poor cell biology in the intrathecal space, to enhance the feasibility and safety of intrathecal MSCs in patients with clinical pain. The hydrogel scaffold has a porous structure that facilitates cell attachment and growth, mimicking the natural ECM of MSC to enhance MSC survival at the target site. The cells embedded in the hydrogel scaffold will not be affected by gravity-induced cell sedimentation, which can increase the retention rate of MSCs at the target site and facilitate the sustained release of MSCs [107]. When selecting hydrogels for intrathecal delivery of MSCs, it is crucial to consider whether they possess the following attributes: excellent biocompatibility, the capacity to sustain cell survival and function, injectability, rapid gel formation and retention at the target site, and the ability for cell release that can degrade in vivo without the need for surgical removal. To date, only a limited number of injectable hydrogels have undergone in vivo safety testing and are deemed suitable for intrathecal cell delivery. Hyaluronic acid (HA) is the main component of the natural ECM and can be safely used to deliver MSCs. Hyaluronan and methylcellulose (HAMC) is a material created by mixing two physical gels, HA and methylcellulose (MC). It is a fast-gelling, injectable material. HAMC has already reached the gelation point before injection. It is injectable due to its shear-thinning property, and its gel strength increases with temperature. It has been proven that HAMC has good biocompatibility in rats and is beneficial for the treatment of spinal cord injury [108–111]. In addition, researchers have developed an injectable biocompatible hydrogel based on manganese, which allows for image-guided cell delivery. The hydrogel was created by blending methacrylated gellan gum (GG-MA) and HA and supplemented with paramagnetic Mn^2+^. MA/HA can serve as a carrier for cell delivery and allows the real-time visualization of hydrogel deposition through T1-weighted magnetic resonance imaging (MRI) [112]. In summary, these engineering strategies combine biomaterials with MSCs to create an effective intrathecal delivery system for pain management.

#### Intranasal delivery

The intranasal route offers several advantages, including rapid absorption, easy dose control, convenient repeated drug delivery, and minimized systemic side effects. In comparison to systemic transvenous delivery, transnasal cell delivery is not impeded by the blood–brain barrier, exhibits superior targeting of the central nervous system, and presents clear advantages in pain management. In a study investigating peripheral neuropathy and pain induced by cisplatin or paclitaxel chemotherapy, intranasally administered MSCs rapidly enter the brain, spinal cord, and meninges in peripheral lymph nodes. MSCs promote the production of IL-10 by macrophages, improving mitochondrial dysfunction in dorsal root ganglion (DRG) neurons, thus significantly reducing mechanical allodynia and spontaneous pain in mice [96]. The intranasal delivery of MSC-EVs has been observed to undergo axonal transport and cerebrospinal fluid circulation through the olfactory and trigeminal pathways, ultimately reaching the olfactory bulb, thalamus, hippocampus, subarachnoid space, and spinal cord in mice and rats [113]. Moreover, nasal delivery offers a non-invasive method for drug administration, which presents a lower risk of tissue injury and infection in comparison to intrathecal injection. The safety profile and patient acceptance of intranasal delivery also surpass those of intrathecal injection. These advantages make intranasal delivery a more attractive approach for delivering MSCs. The effectiveness and safety of intranasal delivery of MSCs have been thoroughly validated in preclinical research on Alzheimer’s disease and Parkinson’s disease [114, 115]. However, there are limitations and challenges present in the clinical application of intranasal delivery of MSCs. First and foremost, the anatomical structure of the human nasal cavity and central nervous system differs significantly from that of rodents. The olfactory epithelium serves as the primary gateway for the entry of cells or drugs into the brain through the nasal cavity. The area, distribution, and permeability of the olfactory epithelium are significantly different between humans and rodents, as well as the absorption rate and deposition dosage of nanoparticles [116]. Secondly, while animal experiments effectively demonstrate the improvement of diseases resulting from intranasal delivery of MSCs, they are unable to provide precise assessments for non-disease indicators such as drooling, sleep, and nocturnal breathing. These indicators are significantly important for evaluating clinical feasibility. These issues indicate that intranasal delivery of MSCs may provide greater advantages compared to other delivery methods in preclinical trials. However, it remains challenging to predict its actual effectiveness and safety in clinical practice. Therefore, future research should collect data from larger animals with anatomies more closely resembling those of humans, as well as evidence from clinical trials. Additionally, further investigation is needed to explore strategies for enhancing the effectiveness of nasal delivery of MSCs.

Some studies use biomaterials to treat nasal mucosa or modify MSCs to increase the delivery rate and targeting of intranasal delivery of MSCs. Some researchers have found that pretreating the olfactory epithelium with hyaluronidase can significantly increase the permeability of the nasal mucosa by prolonging the adhesion time of MSCs to the olfactory epithelium [117]. It is worth noting that studying the permeability of MSCs from the human nasal mucosa in preclinical research can improve the transferability of the results to clinical settings. In another study, the penetrating peptide (Penetratin, P) and rabies virus glycoprotein 29 (RVG29) were embedded into the phospholipid bilayer membrane of the exosome derived from MSCs. The surface-modified exosome was then combined with the biomaterial poly (propylene sulfide)-polyethylene glycol (PPS-PEG) to create an engineered exosome with enhanced penetration capabilities. The P-peptide increased the adhesion and penetration rates of the engineered exocrine in the nasal mucosa, thus effectively increasing the concentration in the brain [114]. The engineered exosomes demonstrate enhanced inhibitory effects on glial cell activation and neuroinflammation, suggesting the potential of this strategy for intranasal MSC delivery in analgesia therapy. In summary, these innovative cell engineering strategies offer potential research directions for optimizing the efficiency of nasal delivery of MSCs. The feasibility of their clinical application still requires further evaluation.

#### Transdermal delivery

Recently, research has explored innovative methods of transdermal delivery of MSCs for the treatment of pain. Microneedles are minimally invasive devices that can penetrate the stratum corneum of the skin without causing pain. They facilitate the delivery of large-molecule drugs, such as DNA, RNA, antibodies, and vaccines, into the skin. Borneol is a substance that can effectively enhance the penetration of drugs through the blood–brain barrier [118]. Some researchers combined exosomes derived from MSCs with borneol-modified liposomes, loading Ziconotide inside, to create microneedles capable of penetrating the skin and the blood–brain barrier. Animal experiments have shown that this transdermal delivery system can transport exosomes to the cerebrospinal fluid through the skin without the need for invasive procedures, such as intrathecal injection [119]. From a clinical perspective, the transdermal delivery system is non-invasive, painless, and well-received by patients, making it a potential new method for delivering MSCs.

In conclusion, all of these innovative studies have provided a promising strategy for MSC engineering. By using cell engineering technology to assemble biomaterials and MSCs together, an effective MSC delivery system can be formed for pain management. It is worth noting that many ongoing clinical trials on intrathecal and intranasal delivery of MSCs have been terminated due to a lack of efficacy and safety. This failure in clinical translation can partly be attributed to the significant differences between experimental animals and human anatomical structures. Therefore, when treating clinical patients with MSCs for pain, the translatability of preclinical research results should be fully considered. Most importantly, when selecting the optimal method for infusing MSCs in patients, it is crucial to comprehensively evaluate factors such as the invasiveness of the delivery method itself, potential safety risks, and the efficiency of MSC delivery. This evaluation should consider the individual characteristics and disease progression of each patient. Consequently, personalized MSC delivery routes should be chosen for each patient.

## Challenges in clinical translation of MSC-based analgesia therapy

### The source and heterogeneity of MSC

MSCs from different sources exhibit unique characteristics and functions. The age, gender, health status, and associated diseases of the MSC donor, as well as the tissue source and isolation method of the MSCs, can all influence the characteristics and analgesic efficacy of MSCs [120]. MSCs derived from younger and healthier donors often exhibit stronger proliferation, differentiation, anti-inflammatory, and immunomodulatory capabilities compared to MSCs obtained from older donors with chronic diseases. They also exhibit stronger resistance to disease environments and greater therapeutic efficacy in treating diseases [121, 122]. A biostatistical analysis investigated the sex-related dimorphism on the hAD-MSCs transcriptome. The data showed that differentially expressed genes between hAD-MSCs sourced from males and females were associated with multiple biological processes such as cell proliferation, migration, differentiation, senescence, immune regulation, and cell communication. Specifically, compared to hAD-MSCs sourced from males, female hAD-MSCs exhibited higher expression of genes involved in cell cycle regulation, such as TFPI2 and GNG11, which were associated with increased susceptibility to cell apoptosis and lower proliferation and migration capacity. In contrast, compared to hAD-MSCs sourced from females, male hAD-MSCs showed lower expression of CXCL3, which may explain their lower adipogenic differentiation ability. Additionally, genes involved in cell–cell or cell-ECM adhesion processes also displayed significant gender differences, suggesting possible different ways of communication between male and female hAD-MSCs [123]. In a comparative study comparing hBM-MSCs and hUC-MSCs in the treatment of neuropathic pain, hBM-MSCs and hUC-MSCs transplantation can relieve neuropathic pain and promote the recovery of motor function after spinal cord injury. Electrophysiological evaluation showed that hUC-MSCs could repair neurons in a large dynamic range better than hBM-MSCs. Moreover, the survival rate of hUC-MSCs was significantly higher than that of hBM-MSCs [124].

Furthermore, it is worth noting that even within the same tissue source of MSCs from the same donor, there is significant heterogeneity within the cell population. Single-cell sequencing of extracted MSCs has revealed the presence of multiple cell clusters with different functional characteristics within MSCs. MSCs from different clusters have distinct surface markers and secrete various proteins [125–127]. Therefore, considering the differences within MSC clusters, utilizing cell sorting techniques to select MSCs with functional characteristics that are most suitable for pain treatment can enhance the efficacy of MSC therapy in pain management.

### Individual differences in recipients and their disease microenvironment

The age of the recipient can affect the distribution and efficacy of MSCs in the body. The tissue and organ distribution of MSCs transplanted intravenously is broader in young mice compared to older mice, indicating that MSCs may be less effective in elderly patients than in younger patients [128]. The inflammatory and oxidative stress microenvironment created by diseases can lead to the rapid aging or death of MSCs that migrate to the site of the disease. Different patients with the same disease may have varying effects on MSCs due to individual differences in their disease microenvironment. This is also one of the important reasons for the differences in efficacy of MSC therapy among different patients.

The gender differences in the mechanisms of pain occurrence are receiving increasing attention from researchers. Microglia is a crucial participant in the mechanism of pain. Studies have found that interventions inhibiting microglia cells can effectively relieve pain in male mice, but the efficacy in female mice is poor. The reason for this phenomenon may be that most previous pain studies only included male animals, and the research results obtained may not accurately represent the situation in female animals. The pain mechanism in female mice may be more related to the role of adaptive immune cells [129, 130]. Therefore, it is encouraged to include both males and females in future preclinical research, and in the process of clinical translation, treatment strategies suitable for male and female pain patients should be developed separately based on gender-specific pain mechanisms.

### Potential safety risks of MSC

Although MSCs have shown great potential for clinical transformation. However, it is important to carefully evaluate the potential risks and adverse reactions associated with their use in patients. It is important to note that the use of MSCs in preclinical and clinical trials for certain diseases has shown potential safety risks, including tumorigenesis and accidental differentiation.

Some studies have reported that MSCs have the risk of tumorigenesis in the treatment of diseases. A study conducted to explore the therapeutic effect of MSCs on myocardial infarction and diabetic neuropathy found that the 4th–8th generation of MSCs cultured in vitro could induce tumor formation after transplantation into the injured site in mice. Further analysis of the chromosomes of tumorigenic MSCs revealed several abnormalities, such as chromosome fusion, breakage, and ring formation. However, tumorigenic MSCs did not show any abnormal morphology or abnormal surface epitopes. The chromosome abnormalities of MSCs may be accumulated in the process of culture and amplification in vitro. However, it is worth noting that chromosome aberrations were detected even in the fourth generation at the early stage of MSC culture. This is usually the minimum passage number required to obtain sufficient MSCs for preclinical or clinical research [131]. Therefore, this study gives us an important hint that it is necessary to closely monitor the chromosome status of MSCs cultured in vitro for disease treatment.

The strong multi-directional differentiation of MSCs enables them to play a repair role in the damaged tissue. However, while the MSCs transplanted into the body differentiate into target tissue cells, they also differentiate into other types of cells. In a study to explore the therapeutic effect of MSCs on myocardial infarction, the researchers injected bone marrow-derived MSCs into infarcted myocardium in mice. Calcification and ossification of the capsule structure were detected 10 days after MSC injection in the infarcted myocardium [132]. This finding reveals the potential risk of using MSCs in myocardial infarction. It is worth noting that since the cellular origin and mechanism of calcification and ossification have not been determined, and mouse data cannot be used to represent human conditions, it is not possible to conclude that MSCs are not suitable for the treatment of myocardial infarction. In another study of age-related macular degeneration, three patients developed severe bilateral vision loss, retinal hemorrhage, and detachment 3–16 days after intravitreal injection of autologous adipose-derived MSCs [133]. The cause of retinal damage induced by MSCs may be related to the differentiation of mesenchymal stem cells into myofibroblasts [134]. These risks of accidental differentiation highlight the importance of monitoring and regulating the differentiation status of mesenchymal stem cells. Numerous studies have focused on finding ways to induce specific differentiation of MSCs to avoid any undesired outcomes arising from accidental differentiation [135, 136]. In the treatment of pain, it is crucial to maintain the cellular stemness of MSCs to ensure their efficacy in pain relief. Therefore, for ongoing clinical trials involving MSCs, reducing the dose of MSCs could lower the risk of tumorigenesis and accidental differentiation. Patients participating in these trials should be closely monitored and followed up over a long period.

## Conclusions and perspectives

Choose an MSC type with functional properties appropriate for treating pain, minimize internal MSC heterogeneity, optimize the MSCs’ in vitro culture environment, and select a personalized MSC delivery method suitable for each patient. These are crucial strategies to enhance the analgesic efficacy of MSCs and important directions for further research. Enhancing the targeted migration of MSCs to the target site in vivo is also a significant research direction. Some studies have developed an engineering strategy to improve the targeted delivery of MSCs to injured sites by modifying the surface of MSCs with an arginine-glycine-aspartic (RGD) peptide. RGD peptide has a strong affinity for integrins, which are widely expressed on the surface of various cells. The interaction between RGD and integrins drives the targeted migration of MSCs towards tubular epithelial cells in acute kidney injury, neovascular endothelial cells in spinal cord injury, and microglia in retinal degeneration, thereby enhancing their therapeutic efficacy [137–139]. Given that integrins are also widely expressed on the surfaces of neurons and glial cells, we speculate whether surface-modified MSCs with RGD can achieve targeted migration in pain treatment. This engineering strategy could potentially enhance the analgesic effect of MSCs by promoting their targeted migration towards activated neurons and glial cells, which is worth exploring, as the overactivation of spinal cord neurons and glial cells is a crucial mechanism of pain.

Considering that chronic pain patients usually take NSAIDs and opioid analgesics, it is worth exploring whether MSC therapy combined with commonly used analgesic drugs can enhance the analgesic effect. At the same time, future research should investigate the interaction between MSC and commonly used analgesic drugs. Whether MSC therapy can reduce the dosage and occurrence of side effects of opioid analgesics is an interesting research direction. It is important to take gender differences into account in future preclinical studies of pain mechanisms and analgesic strategies. The inclusion of experimental animals of different genders in the study will improve the credibility of the research results and promote their clinical transformation.

It should be noted that any strategy that changes the characteristics and efficacy of MSCs will have a profound impact on them. Hence, it is essential to closely monitor the potential adverse effects that may arise from modified MSCs over an extended period. The MSC clinical research guidelines issued by the United States Food and Drug Administration emphasize the need for systematic and thorough safety verification in animal experiments before using MSC therapy in patients [140]. Therefore, research into the modification of MSCs to enhance their efficacy should not only focus on their effectiveness in preclinical research but also pay close attention to the clinical safety and transferability of the modified MSCs. Additionally, the economic cost of large-scale clinical use of modified MSCs and whether it is affordable for patients should also be considered. Currently, there are no established MSC quality control standards to guide MSC production, which is a task that needs to be completed as soon as possible in the future. Despite the challenges in clinical transformation, MSC therapy is a promising strategy for pain management owing to its unique characteristics and is expected to provide better analgesic effects for patients.

## Data Availability

Not applicable.

## Abbreviations

BDNF: Brain-derived neurotrophic factor
CCI: Chronic constriction injury
CCL2: C–C motif chemokine ligand 2
CREB: Cyclic adenosine monophosphate response element-binding protein
CXCL1: C–X–C motif chemokine ligand 1
2D: Two-dimensional
3D: Three-dimensional
DA: Dopamine
DRG: Dorsal root ganglion
ECM: Extracellular matrix
ERK: Extracellular signal-regulated kinase
EVs: Extracellular vesicles
GDNF: Glial cell line-derived neurotrophic factor
GG-MA: Methacrylated gellan gum
GM-CSF: Granulocyte-macrophage colony-stimulating factor
HA: Hyaluronic acid
hAD-MSCs: Human adipose tissue-derived MSCs
hBM-MSCs: Human bone marrow-derived MSCs
hUC-MSCs: Human umbilical cord MSCs
IFN-γ: Interferon-γ
IL-6: Interleukin-6
MAPK: Mitogen-activated protein kinase
MC: Methylcellulose
miRNA: MicroRNA
MMP-9: Matrix metalloproteinase-9
MOR: μ-Opioid receptor
MSCs: Mesenchymal stem cells
MyD88: Myeloid differentiation primary response protein 88
NF-κB: Nuclear factor kappa-B
NGF: Nerve growth factor
NPs: Nanoparticles
NSAIDs: Non-steroidal anti-inflammatory drugs
P2X4R: Purinergic receptor P2X4
PDA: Polydopamine
PKC-γ: Protein kinase C-gamma
REST: Repressor element-1 silencing transcription factor
RGD: Arginine-glycine-aspartic
ROS: Reactive oxygen specie
SCI: Spinal cord injury
STAT3: Signal transducer and activator of transcription 3
TGF-β1: Transforming growth factor-β1
TLR2: Toll-like receptor 2
T-MSC: Tonsil-derived MSC
TNF-α: Tumor necrosis factor alpha
TRAF6: Tumor necrosis factor receptor-associated factor 6
TSG-6: TNF-α-stimulated gene 6
VCAM-1: Vascular cell adhesion molecule-1
VEGF: Vascular endothelial growth factor
